# 802 A retrospective study: rapid removal of biofilm and necrosis with a hygroscopic chemical debriding compound

**DOI:** 10.1093/jbcr/irac012.351

**Published:** 2022-03-23

**Authors:** Michel H Hermans

**Affiliations:** Hermans Medical Consulting, Hoorn, Noord-Holland

## Abstract

**Introduction:**

Removing necrosis and biofilm is an essential step in the treatment of all lesions. A new debriding compound (TDA) uses a hygroscopic mechanism to reach this goal: when in contact with biofilm and necrosis this causes desiccation, with subsequent dissolution of these compounds.

**Methods:**

In a retrospective proof-of-principle study the results of a one-time application of TDA in 54 serious foot and leg lesions, mainly diabetic and venous ulcers, were analyzed.

**Results:**

Outcomes were positive: at study end: 50 out of 54 lesions (92.5%) showed complete granulation and in 40 out of 54 lesions (74.0%) complete reepithelialization was reached.

TDA efficacy is represented by three cases. In the first one, an 83-year-old diabetic female with sever peripheral arterial disease and status post-toes-amputation suffered from a gangrenous lesion of the left foot. A second case involved a 77-year-old female who had a non-healing, post-trauma lesion over the Achilles tendon, and the last case entails an 81-year-old male with a non-healing lesion on the left lower leg. All patients showed very rapid debridement and subsequent healing and reepithelialization

**Conclusions:**

Typically, diabetic, and venous ulcers require repeated debridement and treatment of the biofilm is notoriously difficult. It also takes a long time before the woundbed is healthy enough to start to granulate and, subsequently, epithelialize. The results, obtained in this study, indicate that a one-time application of TDA is highly effective for “jumpstarting” wound healing.

